# Successful conservative management with methotrexate and mifepristone of cervical pregnancy

**DOI:** 10.1016/S1674-8301(11)60009-2

**Published:** 2011-01

**Authors:** Eliza Shrestha, Yuebo Yang, Xiaomao Li, Yu Zhang

**Affiliations:** Department of Obsterics and Gynecology, the Third Affiliated Hospital of Sun Yat-sen University, Guangzhou, Guangdong 510630, China

**Keywords:** cervical pregnancy, ectopic pregnancy, conservative treatment

## Abstract

This study investigated possible effective treatments for cervical pregnancy, a rare form of ectopic pregnancy. The clinical records of 11 cases of ectopic pregnancy admitted to the Third Affiliated Hospital of Sun Yat-sen University from 1998 to 2010 were analyzed. All patients were treated with intermuscular injection of methotrexate (MTX, 50 mg), and oral mifepristone (25 mg, bid). All cases were successfully cured by conservative treatments using methotrexate plus mifepristone. Cervical pregnancy is a contributive factor to mutiple abortions and curettages. Methotrexate plus mifepristone, curettage through hysteroscopy and intracervical obturation with gauze are effective treatments of cervical pregnancy without the need for surgical intervention.

## INTRODUCTION

Cervical pregnancy, a rare form of ectopic pregnancy, has a prevalence of 0.1% among pregnancies.[1] The diagnosis of cervical pregnancy is clinically challenging and commonly delayed. Although the advent of ultrasonography has facilitated more accurate diagnosis, problems still exist in developing countries where access to this technology is still limited. Until recently, hysterectomy was performed as a treatment because of profuse life-threatening bleeding. However, early diagnosis with ultrasound, and promising medical therapy have improved the outcome of conservative nonsurgical management of this entity. In this report, we described a retrospective study of 11 cases of ectopic pregnancy admitted in the Third Affiliated Hospital of Sun Yat-sen University from 1998 to 2010. We briefly summarized the risk factors of cervical pregnancy and the effective treatments of cervical pregnancy without the need for surgical intervention.

## CLINICAL DATA AND RESULTS

The median age of 11 women was 31.5 years (ranging from 21 to 42). One patient had her first pregnancy at the age of 36 years, while the remaining 10 patients were multipions (2 to 4 times) with an average of 2 multiparous.

All patients complained of irregular vaginal bleeding when they were seen at the Emergency Department. Three cases had a history of abdominal pain, and 8 cases had no history of abdominal pain. Two cases had heavy vaginal bleeding of about 1,000 mL during their hospital visit, while 9 cases had slight vaginal bleeding. All 11 cases had a history of amenorrhea for 34 to 84 d with an average of 53.1 d. Eight patients had no obvious enlargement of the uterus but the cervix was significantly enlarged. Three cases had enlargement of both the uterus and cervix.

The blood of all patients was quantitatively analyzed for serum β-human chorionic gonadotrophin (HCG) level at the time of admission, which ranged between 1,509.0-79,192.0 mU/mL, with an average of 20,369.05 mU/mL. After drug treatment, serum β-HCG levels decreased to 15.7-542.0 mU/mL, and the average was 178.6 mU/mL.

All patients underwent B-ultrasound (BUS) examination (11/11). Nine cases revealed a cervical pregnancy (9/11), and 2 cases underwent color doppler ultrasonography to confirm the diagnosis of cervical pregnancy (2/11). Nine cases underwent cervical scrapings (9/11), and 2 cases underwent simple hysterectomy (2/11). Specimen was sent to a pathologist for histological diagnosis of cervical pregnancy. One case showed a β-HCG drop to 151 mU/mL and BUS showed that the cervical canal had abnormal echoes about 2.3 cm×2.4 cm×2.5 cm in size. Curettage was done but no discharge was seen. Out-patient follow-up was recommended, and the patient had a subsequent regular menstruation cycle (***Table 1***).

**Table 1:** Clinical Characteristics

Index	
Age (years)	21-42
	31.5 (Median)
β-HCG (mU/mL)	1,509.0-7,912.0
	20,369.05 (Average)
BUS	9 cases
Ultrasonography color doppler	2 cases
Cervical scraping	9 cases
Simple hysterectomy	2 cases
Liver toxicity	2 cases
Hysterescopic electrocaogulation	3 cases

β-HCG: β-human chorionic gonadotrophin; BUS: B-ultrasonography.

Treatment methods for all patients who were hospitalized included intramuscular (IM) injection of methotrexate (MTX, 50 mg), and oral mifepristone (25 mg, bid). Serum β-HCG level was checked every 3 d, and liver function was tested weekly. At the same time, vital signs of all patients were monitored. All treatment protocols were carried out with the consent of patients or the patients' family members.

All patients after IM injection of MTX combined with oral mifepristone showed a progressive decline in their blood β-HCG levels (11/11). Two patients had mild liver toxicity (2/11). After treatment, liver function tests were within normal limits. Two cases were discharged without curettage (2/11). BUS of 3 cases revealed an abnormal echo at the endocervical canal ranging from 5 cm×5 cm×4 cm to 8 cm×7 cm×7 cm in size. Hysterescopic electrocaogulation was done after curattege to stop the bleeding and then finally followed with gauze packing (3/11). To preserve the uterus, 1 case underwent BUS guided uterine aspiration (1/11). Two cases with 50 d gestation exhibited vaginal bleeding of about 1,000 mL at the time of admission. After terminated pregnancy by drugs, serum β-HCG decreased to 288 mU/mL. BUS revealed an abnormal echo 7 cm×6 cm×6 cm in size at the endocervical canal. Total hysterectomy was then performed (2/11) with the consent of the patients.

## DISCUSSION

As cervical pregnancy is an infrequent clinical incident, no authoritative or randomized controlled trial exists. Cervical pregnancy was first described in 1817 and first named as such in 1860. It accounts for about 0.1% of pregnancies[1],[2].

Cervical pregnancy has been classified as a distal ectopic pregnancy. There are several theories to explain the etiology. According to Clarke[2], a delay in the rate of travel or an increase in the rate of ripening of the fertilized ovum leads to a proximal ectopic pregnancy, whereas an increased rate of travel or decreased rate of ripening leads to a distal ectopic pregnancy. Several authors believe that an antecedent abortion is etiologically related to a subsequent cervical pregnancy[3]–[6]. There are many predisposing factors for cervical pregnancy such as endocrine disorders, or uterine abnormalities from uterine cancer to the uterine cavity distortion, *in vitro* fertilization[7], dilatation and curettage, uterine scaring, pelvic inflammatory disease, leiomyoma, intrauterine devices and a primary embryo anomaly. Despite these speculations, the cause of cervical pregnancy still remains unknown[8]. In our study, among the 11 patients, 10 patients had a history of abortion or a history of cesarean section and 1 case was pregnant for the first time at an old age, which suggests that patient age may be related to endometrial dysplasia, endocrine disorders and other factors. A history of abortion or curettage is closely associated with cervical pregnancy, and awareness about women's reproductive health education issues to reduce abortion rates could reduce the incidence of cervical pregnancy and maternal mortality.

In the majority of the cases of cervical pregnancy, the main symptoms are vaginal bleeding or bloody discharge from a low volume to heavy volume, which is not associated with abdominal pain. The main signs are enlarged and bulky cervix, occasionally larger than the fundus, forming the so-called hourglass uterus. A visible cervical lesion, often described as blue or purple and distended or edematous in appearance may be noted. Preoperative detailed history, physical examination, BUS, and serum β-HCG determination will help with early diagnosis of cervical pregnancy. None of these signs are either diagnostic or invariably present, however, and if the suspicion of cervical pregnancy arises, radiological evaluation is mandatory as BUS showed a cervical pregnancy in our 9 cases. Only 2 cases needed to undergo colour doppler ultrasonography. In 1978, Raskin[9] suggested that the diagnosis by BUS examination of cervical pregnancy required four factors: enlargement of the cervix, uterine enlargement, diffuse amorphous intrauterine echoes and absence of intrauterine pregnancy.

The cervix is composed of fibrous connective tissue, with only 15% being comprised of smooth muscle component. Therefore, it is difficult to close the opening of sinusoids when bleeding is heavy. Oxytocin preparations have no effect on the hemorrhage since the cervix is refractory to them. Thus, in the majority of cases, definitive treatment is abdominal hysterectomy. Patients with early cervical pregnancy before 12 w of gestation are also more likely to have a successful treatment because there is less trophoblastic infiltration into the cervical wall. Farabow *et al.*[10] were the first to report the use of MTX for cervical pregnancy. MTX is a folic acid antagonist which acts as a suppressor of trophoblast cell division during pregnancy, thus causing the nutrient cell to die. In the presence of progesterone, mifepristone acts as a competitive receptor antagonist at the progesterone receptor. Mifepristone induces decidual breakdown indirectly, leading to trophoblast detachment and resulting in decreased syncytiotrophoblast production of HCG, which in turn causes decreased production of progesterone by the corpus luteum. The combination of these two drugs will prevent the development of embryos and speed up embryonic death, which will definitely shorten the treatment time. Moreover, the combination of these two drugs has benefits. In our case, combination of mifepristone reduced the dose of MTX, which meant reduction of toxicity as methotrexate is a cytotoxic drug. During the treatment, it is necessary to monitor blood β-HCG levels and to know the condition of the fetal tissue absorption in the endocervical canal by BUS. At the same time, liver and renal function tests should be done to determine development of toxicity. After the combination therapy of MTX plus mifepristone, chorionic villi necrosis following embryonic death leads to decreased blood loss during curettage. As embryo implantation sites are seen more clearly during hysteroscopy and curettage, it is easier to coagulate the bleeding site. Therefore, this protocol provides a complete treatment for cervical pregnancy. In our study, 11 patients received the combined treatment of MTX plus mifepristone, which led to a significant decrease of blood HCG level. Since 3 cases had an enlarged and bulky cervix, we performed hysteroscopy and curettage under direct visualization of the endocervical canal, and gauze packing was used to apply direct pressure on the bleeding site, achieving good results.

In conclusion, the key to successful conservative management of a cervical pregnancy is essentially an early diagnosis and early treament. Options for treatment of cervical ectopic pregnancy depend on the fetal gestational age, the woman's desire to maintain fertility, and relative quality of life. MTX combined with mifepristone, hysteroscopy and curettage under direct visualization and cervical gauze packing is an effective treatment of early cervical pregnancy.
